# Self-Management education for adults with poorly controlled epILEpsy (SMILE (UK)): a randomised controlled trial protocol

**DOI:** 10.1186/1471-2377-14-69

**Published:** 2014-04-03

**Authors:** Ines Kralj-Hans, Laura H Goldstein, Adam J Noble, Sabine Landau, Nicholas Magill, Paul McCrone, Gus Baker, Myfanwy Morgan, Mark Richardson, Stephanie Taylor, Leone Ridsdale

**Affiliations:** 1 Department of Clinical Neuroscience PO 43, Institute of Psychiatry, King’s College London, Denmark Hill Campus, London SE5 8AF, UK; 2 Department of Psychology PO 77, Institute of Psychiatry, King’s College London, Denmark Hill Campus, London SE5 8AF, UK; 3 Department of Psychological Sciences, Institute of Psychology, Health & Society, The Whelan Building, University of Liverpool, Liverpool L69 3GL, UK; 4 Department of Biostatistics PO 20, Institute of Psychiatry, King’s College London, Denmark Hill Campus, London SE5 8AF, UK; 5 Department of Health Service & Population Research PO 24, Institute of Psychiatry, King’s College London, Denmark Hill Campus, London SE5 8AF, UK; 6 Department of Clinical Pharmacology, University of Liverpool, Liverpool L69 3 BX, UK; 7 Division of Health and Social Care Research, School of Medicine, King’s College London, 7th Floor Capital House, 42 Weston Street, London SE1 3QD, UK; 8 Barts & The London School of Medicine and Dentistry, Centre for Health Sciences Blizard Institute, Abernethy Building, 2 Newark Street, London E1 2AT, UK

**Keywords:** Epilepsy, Seizures, Self-management education, Clinical trial, Quality of life

## Abstract

**Background:**

Teaching people with epilepsy to identify and manage seizure triggers, implement strategies to remember to take antiepileptic drugs, implement precautions to minimize risks during seizures, tell others what to do during a seizure and learn what to do during recovery may lead to better self-management. No teaching programme exists for adults with epilepsy in the United Kingdom although a number of surveys have shown patients want more information.

**Methods/Design:**

This is a multicentre, pragmatic, parallel group randomised controlled trial to evaluate the effectiveness and cost-effectiveness of a two-day Self-Management education for epILEpsy (SMILE (UK)), which was originally developed in Germany (MOSES).

Four hundred and twenty eight adult patients who attended specialist epilepsy outpatient clinics at 15 NHS participating sites in the previous 12 months, and who fulfil other eligibility criteria will be randomised to receive the intervention (SMILE (UK) course with treatment as usual- TAU) or to have TAU only (control). The primary outcome is the effect on patient reported quality of life (QoL). Secondary outcomes are seizure frequency and psychological distress (anxiety and depression), perceived impact of epilepsy, adherence to medication, management of adverse effects from medication, and improved self-efficacy in management (mastery/control) of epilepsy.

Within the trial there will be a nested qualitative study to explore users’ views of the intervention, including barriers to participation and the perceived benefits of the intervention. The cost-effectiveness of the intervention will also be assessed.

**Discussion:**

This study will provide quantitative and qualitative evidence of the impact of a structured self management programme on quality of life and other aspects of clinical and cost effectiveness in adults with poorly controlled epilepsy.

**Trial registration:**

Current Controlled Trials: ISRCTN57937389.

## Background

Epilepsy is a long-term neurological condition and the most common serious disorder of the brain [1]. Approximately 1% of the UK population have diagnosed epilepsy [2] and following diagnosis, approximately 40% of the patients will continue to experience two or more seizures each year [3]. These people are at higher risk of suffering injury, premature death, as well as experiencing psychological distress and perceived stigmatization due to their condition [2,4]. Poorly controlled epilepsy is also costly to society. In the EU, the total cost of epilepsy was estimated to be €15.5 billion in 2004, the total cost per case was €2000 - €11500 [5]. One way in which costs to society are felt is through the costs of providing emergency care to people with epilepsy (PWE), some of whom attend repeatedly [6,7]. Six out of seven admissions for epilepsy are on an emergency basis [7] and of neurological conditions epilepsy is associated with the highest rate of emergency readmissions within the same year [8,9]. There are also important indirect social costs through lost/absent employment [10].

Coping with life in the context of epilepsy requires people with epilepsy to become confident with managing their own condition [10,11]. As well as needing to accept a potentially stigmatizing diagnosis, they may need to learn to identify and manage triggers for seizures within their surroundings, implement strategies to remember to take a number of antiepileptic drugs (AEDs), implement precautions to minimize risks due to seizures, tell others what to do when a seizure occurs and learn what to do during recovery [12-14].

NHS policy is to empower and support people with long-term conditions to understand their own needs and self-manage them [15]. Routine group education programmes for people with chronic conditions, such as diabetes and arthritis are already implemented by the NHS (e.g. DAFNE [16], DESMOND [17]), and there is a need for a similar programme for people with epilepsy. A consistent finding in surveys of people with epilepsy is that they want better provision of information about how to live with and manage their epilepsy [18-21]. One survey of patients with poorly controlled epilepsy found that 1/3rd reported not being told what epilepsy was, over 90% wanted more information about the disease, and ~75% felt they had not been given enough information about the side effects of antiepileptic drugs (AEDSs) [18]. Over 60% wanted to talk to someone other than a consultant about epilepsy. Dawkins and colleagues [22] found that patients with epilepsy knew no more about the disorder than those without epilepsy.

Cochrane reviews have identified four educational interventions for people with epilepsy from around the world [23,24]. None of these interventions had been tested in the UK, but one of the interventions has been more robustly evaluated and shows promise for use in the UK. This programme, developed iteratively in Germany with the involvement of people with epilepsy is called ‘*Mo*dular *S*ervice Package *E*pilep*s*y’ (MOSES) [25,26]. The original randomised controlled trial of MOSES included people with poorly controlled epilepsy, 72% of whom had more than 12 seizures in the previous six months. The reported benefits of MOSES included improved knowledge about epilepsy, better seizure control and coping and greater tolerance of and fewer reported AED side effects.

MOSES has been developed and trialled in Germany, Austria and Switzerland and translated into English [27]. With input from British Epilepsy Association, we have modified it for use in the UK, and will employ a randomised controlled trial to test whether it affects quality of life, clinical outcomes and cost effectiveness of health service use compared to treatment as usual (TAU).

Advisors from Epilepsy Action contributed to the design of the intervention, helping to identify appropriate outcome measures and directing us to adopt a waiting list control design.

### Objective

SMILE (UK) is a complex intervention [28] with a range of training modules. We have identified three main knowledge gaps which we plan to address in the randomised controlled trial:

1) the acceptability and appropriateness of its format in an outpatient UK NHS setting;

2) its effectiveness in improving QoL for people with poorly controlled epilepsy;

3) its cost-effectiveness.

## Methods and design

### Trial design

This is a multicentre pragmatic parallel group randomised controlled trial with 1:1 randomisation (intervention: control). The patients are followed up for 12 months.

### Trial settings

The trial will take place in London and other parts of South-East England. We have recruited 15 NHS neurologists who specialise in epilepsy and whose clinic lists will be used to identify potential study participants.

### Target population

We plan to recruit adults with epilepsy aged 16 years and over who attended neurology outpatient appointments in the previous 12 months, and who satisfy study eligibility criteria.

Inclusion criteria:

• Have a documented diagnosis of epilepsy (all epilepsy syndromes and seizures types permitted)

• Are currently being prescribed AEDs

• Are aged ≥16 years (no upper age limit)

• Are able to provide informed consent, participate in the workshops and complete the questionnaires in English

• Have had at least 2 seizures in previous 12 months (as reported by patient)

Exclusion criteria:

• Have actual/suspected psychogenic non-epileptic seizures only

• Have acute symptomatic seizures related to acute neurological illness or substance misuse

• Have a severe psychiatric disorder (e.g., psychosis) or terminal medical condition

• Are enrolled in other epilepsy-related non-pharmacological treatment studies

### Participant recruitment

Patients attending neurology outpatient clinics within the preceding 12 months will be identified by Local Investigators (neurologists) from electronic medical records at the participating NHS Trusts.

Each patient will receive a letter from the consultant neurologist responsible for their medical care. This letter will inform patients of the study and give them an opportunity to opt-out of the next step, the screening of their medical records to evaluate eligibility criteria, within three weeks.

Following completion of medical records screening at each study site, the patients identified as potentially eligible will be sent a second letter from the consultant, inviting them to take part. Patients who are not interested in participating are asked to return the opt-out reply slip within three weeks of the receipt of the invitation. Patients who do not opt-out at this stage will be contacted by phone by one of the research workers.

This telephone call is an opportunity to further explain the study, confirm eligibility and to arrange an appointment for obtaining consent and collecting data for baseline (pre-randomisation) measures. The participants are provided with a patient information sheet and a free post envelope with each of the letters.

Over the course of the study the participants are required to complete three sets of questionnaires (Table 1) either in a face-to-face interview with a research worker (at baseline and at 12-month follow-up) or through postal return of the completed questionnaire (at 6-month follow-up). In order to encourage continued participation in the study we will offer each participant a £20 voucher upon completion of the 12 month questionnaire (end of follow-up period).

**Table 1:** Outcome measures and data collection

**Outcome variables**	**Measures**	**No. items**	**T0**	**T1**	**T2**
**Primary outcome**					
Quality of life	QOLIE-31-P [29]	39	RW*	SR**	RW
**Secondary outcomes**					
Seizure frequency	Two scales [30,31]	2	RW	SR	RW
Seizure recency	Patient reported date of last seizure	1	RW	SR	RW
Impact of epilepsy	Impact of epilepsy scale [32]	9	RW	SR	RW
Medication adherence	Epilepsy Self Management Scale [33]	10	RW		RW
Medication adverse effects	QOLIE-31-P [29]	2	RW		RW
Psychological distress	Hospital Anxiety and Depression scale [34,35]	14	RW		RW
Perceived stigma	Stigma of Epilepsy Scale [36]	3	RW		RW
Mastery/control of epilepsy	Epilepsy-specific scale [37]	6	RW		RW
Health economics	Client Service Receipt Inventory [38] and EQ-5D [39]	13	RW		RW

*RW = research worker collected data (in a face to face interview with a participant).

**SR = self reported data (questionnaires completed by the participant and posted to the research team).

**Figure 1:**
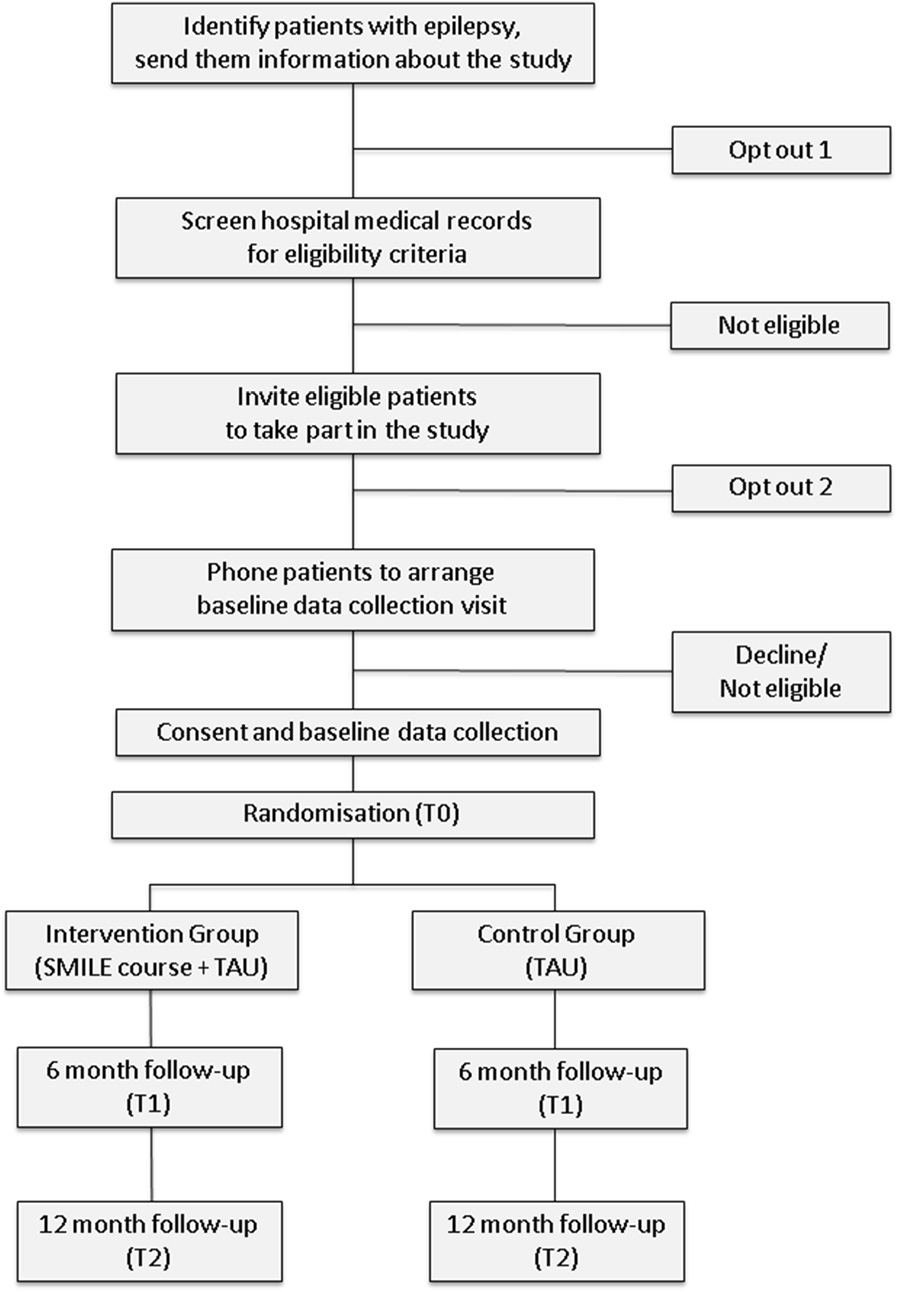
Study flow diagram.

The participants and data flow in the study are shown in Figure 1.

### Consent

The research workers will visit potential participants at their home, or at a place of their choice and explain the study in detail. Those who agree to participate and satisfy eligibility criteria will sign the consent form and complete a set of questionnaires before being randomised.

### Intervention

The intervention is a group-based, interactive course. It is delivered by two health professionals (educational facilitators, EFs) to groups of 8–12 participants (who may include carers of PWE attending the group). The intervention consists of interactive discussion, presentation slides, the use of flip-charts and a workbook. The workbook serves as a source of information and provides space for note-taking and exercises completed during the sessions. There are nine modules in the course: 1. Living with epilepsy; 2. People with epilepsy; 3. Basic knowledge about seizures; 4. Diagnosis; 5. Treatment; 6. Self control; 7. Prognosis ; 8. Personal and social life; 9. Network epilepsy. Delivery of all the modules takes approximately 16 hours over two consecutive days.

The courses will be delivered at the participating NHS sites. Attendance of the course and any interruptions (e.g. due to seizures) will be monitored, and reported through treatment attendance logs. EFs will record information about seizures that occur at the time of treatment, because they may impact on how much treatment the patient received during the two day course.

SMILE (UK) courses will be offered to those randomised to the control group (TAU) once all the follow-up data has been collected.

### Educational facilitators

The courses will be delivered by teams of two EFs who are NHS-employed healthcare professionals (epilepsy nurse specialists or clinical physiologists). They (n = 12) underwent a standardised two-day training course delivered by a team of MOSES specialists from Germany (see Acknowledgements). Four of the health professionals who received the training then delivered two sessions of SMILE (UK) courses to patients with epilepsy who volunteered to take part in the pilot study (through Epilepsy Action). Based on the feedback from the volunteers and from observers of the pilot courses, the research team provided review seminars to all the trained EFs to re-enforce the skills and strategies necessary for successful delivery of the courses.

### Treatment fidelity

A checklist of treatment components based on the finalised course structure and content will be devised following consensus among lead co-investigators in this area. A rating scale will be developed and piloted on four randomly-selected courses to assess variability across raters, clarify the meaning of individual items, and improve coding rules and future inter-observer reliability.

With the permission of the participants, all SMILE (UK) courses will be audio-recorded to enable two independent researchers to identify the presence of treatment components within the course and to determine whether specific modules were appropriately delivered. The aim is for two researchers to rate 25% of course sessions independently.

Courses for treatment integrity rating will be selected randomly (using computer generated numbers) stratified by the EF teams and by the time they took place (early, late or in the middle of the trial). The EFs will not be informed about which sessions will be selected for rating.

### Outcomes and outcome measures

#### Primary outcome

The primary objective of the SMILE (UK) trial is to assess whether participation in the course leads to a change in QoL score in people with poorly controlled epilepsy. This outcome will be measured using the ‘Quality of Life In Epilepsy 31 P’ scale (QOLIE-31-P) [29]. This questionnaire will be completed by the participants prior to randomisation (2–3 weeks) and at 12 months following randomisation in a face-to-face interaction with a research worker. At 6 months post randomisation, the questionnaire will be mailed to participants for completion.

#### Secondary outcomes

Secondary objectives are to assess whether SMILE (UK) course participation leads to: reductions in seizure frequency and psychological distress (anxiety and depression), and improvements in perceived impact of epilepsy, adherence to medication, management of adverse effects from medication, and self efficacy in management (mastery/control) of epilepsy, and whether it is cost-effective. All the outcomes will be measured using the instruments shown in Table 1. Completion of the questionnaires is estimated to take about one hour of the patient’s time.

At 6 months after randomisation a subset of instruments will be posted to the participants for completion on their own. To encourage return of completed questionnaires a phone call to the participant will be scheduled approximately a week after posting them.

### Other data

Demographic information (age, sex, ethnicity, educational background, living arrangements, marital status) will be collected prior to randomisation as part of a face-to-face interview with a research worker. Details regarding any newly diagnosed conditions or symptoms experienced by the study participants since randomisation, which may be interpreted as adverse events, will be collected at 6 and 12 months post-randomisation.

### Sample size calculations

The sample size calculation was based on the primary outcome measure, QoL. The primary intention-to-treat (ITT) analyses will compare two equally-sized treatment arms, treatment and control, on the QOLIE-31-P scale at 12 months. An overall sample size of N = 320 (randomised 1:1) provides 91.3% power to detect an effect size of d = 0.4 on the QOLIE-31-P using an analysis of covariance with 2-sided 5% significance tests. This calculation is based on the conservative assumption of a zero correlation between baseline and post treatment scores on QOLIE-31-P. An effect size of d = 0.4 corresponds to a change of around 6–7 points on the overall QoL score. Since the active treatment is a group treatment, delivered by different therapists within sites, we will allow for standard error inflation due to training group effects. We estimate that attrition rate at 1 year will be around 25%. Therefore, to ensure adequate and equally sized groups an initial sample of N = 428 patients is required (n = 320/0.75; 214 patients per arm).

### Randomisation and concealment

Randomisation will be carried out remotely by the King’s Clinical Trials Unit (KCTU) at the Institute of Psychiatry (http://www.ctu.co.uk) following consent and completion of the baseline data collection. The unit of randomisation will be the individual participant and randomisation will be in 1:1 ratio between the intervention and the control group, stratified by the location of epilepsy clinics from which the patients were recruited. The results of the allocation will be concealed from the trial statistician and the research workers responsible for consent and data collection. The trial manager receives notification of outcomes of randomisation.

### Statistical analyses

The objective of the statistical analyses is the evaluation of the effectiveness of the SMILE intervention. The ITT approach, which analyses patients in the groups to which they were randomised irrespective of their treatment compliance, will be used throughout to estimate effectiveness. Confounding bias and systematic measurement error will be avoided by the use of randomisation and blinding of outcome assessors respectively.

Linear mixed modelling (LMM) will be employed to estimate the primary outcome difference between the trial arms (effectiveness). Models will contain the following fixed effects: the group difference of interest - modelled by an effect of intervention arm, time (6 m or 12 m) and an interaction term - and dummy variables representing the randomisation stratifier (up to 15 treatment centres). To account for correlations between repeated measures on the same individual, a subject-varying random intercept will be included. To allow for correlations between attendees of the same course group a further random effect that varies with training group will be allowed for within the intervention arm. Treatment effects on secondary outcomes will be assessed similarly, using generalisations of the linear mixed model to allow for non-normal data where necessary (e.g. time elapsed since last seizure or seizure frequency).

There will be missing data in post treatment outcome variables where participants are lost to follow-up. The LMM analyses are based on maximum likelihood and resulting inferences are valid provided the missing data generating mechanism is missing at random (MAR). We will empirically assess whether any baseline variables (e.g. age, gender, age when epilepsy diagnosed) predict missingness and if so, we would condition on such variables by including them in the statistical model. We will also assess whether missingness is related to post treatment variables (e.g. other outcomes and most notably non-compliance with the intervention). Should this be the case we will use multiple imputation to generate inferences that are valid under this type of MAR [40]. However, since any analysis will only be valid under MAR and not if the data generating process is informative, a formal sensitivity analysis [41] will assess the impact on the treatment effect estimate of any likely departures from MAR.

If there is considerable non-compliance with the SMILE (UK) training programme we will carry out further explanatory analyses to assess the efficacy of the treatment. Specifically, we will employ instrumental variable methods to evaluate the causal effect of receiving the intervention in the subpopulations of participants who would receive a ‘high dose’ or a ‘high quality’ version of intervention if offered. We call this subpopulation the “compliers” and will evaluate efficacy by estimating the complier average causal effect of the intervention. We expect the randomisation itself to serve as a strong instrumental variable for this purpose [42].

### Cost-effectiveness analysis

Intervention costs will be estimated based on staff time required for training, supervision and delivery, overheads and capital costs combined with attendance data to estimate the cost per participant. Other costs will be calculated by combining service use data with unit cost information [43]. Lost employment costs will be calculated by combining lost work days with average wage rates. Health care costs and societal (including informal care and lost employment) costs will be compared between the two arms. Cost data are usually skewed and we will use bootstrap methods to produce 95% confidence intervals around the cost differences. Cost-effectiveness will be assessed using health care and societal perspectives by combining the costs with data on the primary outcome measure at 12 months. Cost-utility will be measured by combining costs with quality-adjusted life years (QALYs), derived from the European Quality of Life-5 Dimensions (EQ-5D) questionnaire. If the intervention results in better outcomes and lower costs, then it will be ‘dominant’. If, though, it results in better outcomes but higher costs, then incremental cost-effectiveness ratios will be calculated which will indicate the extra cost incurred to achieve a one-unit improvement on the QOLIE or one extra QALY (both at 12 months). To address the uncertainty around these estimates 1000 cost-outcome combinations will be produced using bootstrap methods and plotted on a cost-effectiveness plane. Cost-effectiveness will also be interpreted using cost-effectiveness acceptability curves, which will show the probability that the intervention is the most cost-effective option for a range of different values placed on an improvement in outcome. The range of values for QALYs will be £0 to £100,000; this includes the threshold that appears to be used by the U.K.’s National Institute for Health and Clinical Excellence when judging the cost-effectiveness of a health technology. The range for improvements on the QOLIE will be chosen such that values at which the intervention or TAU has a 50% and 70% and 90% likelihood of being cost-effective are identified.

### Nested qualitative study

Individual in-depth interviews will be conducted with 20 study participants who were randomised to SMILE (UK) intervention and their main supporter(s) with whom they have regular contact. Purposive sampling will include participants who completed the trial and those who did not, and carers who attended SMILE (UK) sessions. We will aim to ensure that the sample represents differences in gender, age, ethnicity and severity of seizures as recorded at the beginning of the trial.

Interviews will take place at selected participants’ homes or a convenient public place if preferred. In a guided conversation participants will be asked to describe their experiences in taking part in the courses, their perceptions of things they valued and found of particular benefit for them as well as negative aspects, and will also discuss any factors that encouraged or hindered their participation in the courses, and whether and in what ways they have continued to make use of the training received. The content of the course workbook will also be discussed in relation to participants’ own needs and lifestyles.

The interviews will be audio-recorded, transcribed and textual coding undertaken. The lower level codes will be grouped into themes and analysis undertaken through a process of constant comparison, with particular attention given to the analysis and explanation of variations between respondents and the views of study participants and carers. Two members of the research team will participate in data analysis to reduce bias in the identification and interpretation of themes.

### Monitoring

Monitoring of this trial by the Trial Steering (TSC) and Data Monitoring Committees (DMEC) will help to ensure that the objectives of the trial are reached as planned. Compliance with Good Clinical Practice and scientific integrity will be managed by the study management team (includes co-investigators, trial manager and KCTU) through regular and ad-hoc meetings. The KCTU will provide regular reports on data quality to ensure the integrity of randomisation, to monitor the level of missing data and the timeliness of data entry and check for illogical or inconsistent data. Data collection procedures will be monitored and source data verification against the paper data collection forms undertaken at regular intervals.

## Discussion

This is the first randomized controlled trial of self-management education for adults with poorly controlled epilepsy in the UK. The intervention has been adapted for use within the NHS and the study aims to provide qualitative and quantitative evidence of the impact of a complex intervention on patients in terms of its clinical and cost effectiveness.

### Ethics approval

NRES Committee London – Fulham, reference number 12/LO/1962.

### Trial status

Ongoing (recruiting participants).

## Competing interests

The authors received a contribution from Sanofi UK to enable publishing of the patient workbook. There are no other competing interests.

## Authors’ contributions

LR responded to the NIHR call and recruited the collaborators on the study. LR, LG and AN wrote the protocol with feedback from SJCT, SL, PMcC, MR and GB. NM and MM joined the study group and contributed importantly with respect to quantitative and qualitative methodology respectively. IKH (the Trial Manager) wrote the first draft of this manuscript. All authors contributed to subsequent revisions and approved the content.

## Pre-publication history

The pre-publication history for this paper can be accessed here:

http://www.biomedcentral.com/1471-2377/14/69/prepub
